# Improving total saccharification yield of Arabidopsis plants by vessel-specific complementation of *caffeoyl shikimate esterase* (*cse*) mutants

**DOI:** 10.1186/s13068-016-0551-9

**Published:** 2016-07-07

**Authors:** Lívia Vargas, Igor Cesarino, Ruben Vanholme, Wannes Voorend, Marina de Lyra Soriano Saleme, Kris Morreel, Wout Boerjan

**Affiliations:** Department of Plant Systems Biology, VIB, 9052 Ghent, Belgium; Department of Plant Biotechnology and Bioinformatics, Ghent University, 9052 Ghent, Belgium; Department of Botany, Institute of Biosciences, University of São Paulo, Butantã, SP Brazil

**Keywords:** *Arabidopsis thaliana*, Caffeoyl shikimate esterase (CSE), Genetic engineering, Lignin, Saccharification, Secondary cell wall, Vessel-specific complementation

## Abstract

**Background:**

Caffeoyl shikimate esterase (CSE) was recently characterized as an enzyme central to the lignin biosynthetic pathway in *Arabidopsis thaliana*. The *cse*-*2* loss-of-function mutant shows a typical phenotype of lignin-deficient mutants, including collapsed vessels, reduced lignin content, and lignin compositional shift, in addition to a fourfold increase in cellulose-to-glucose conversion when compared to the wild type. However, this mutant exhibits a substantial developmental arrest, which might outweigh the gains in fermentable sugar yield. To restore its normal growth and further improve its saccharification yield, we investigated a possible cause for the yield penalty of the *cse*-*2* mutant. Furthermore, we evaluated whether *CSE* expression is under the same multi-leveled transcriptional regulatory network as other lignin biosynthetic genes and analyzed the transcriptional responses of the phenylpropanoid pathway upon disruption of *CSE*.

**Results:**

Transactivation analysis demonstrated that only second-level MYB master switches (MYB46 and MYB83) and lignin-specific activators (MYB63 and MYB85), but not top-level NAC master switches or other downstream transcription factors, effectively activate the *CSE* promoter in our protoplast-based system. The *cse*-*2* mutant exhibited transcriptional repression of genes upstream of *CSE*, while downstream genes were mainly unaffected, indicating transcriptional feedback of *CSE* loss-of-function on monolignol biosynthetic genes. In addition, we found that the expression of *CSE* under the control of the vessel-specific *VND7* promoter in the *cse*-*2* background restored the vasculature integrity resulting in improved growth parameters, while the overall lignin content remained relatively low. Thus, by restoring the vascular integrity and biomass parameters of *cse*-*2*, we further improved glucose release per plant without pretreatment, with an increase of up to 36 % compared to the *cse*-*2* mutant and up to 154 % compared to the wild type.

**Conclusions:**

Our results contribute to a better understanding of how the expression of *CSE* is regulated by secondary wall-associated transcription factors and how the expression of lignin genes is affected upon *CSE* loss-of-function in Arabidopsis. Moreover, we found evidence that vasculature collapse is underlying the yield penalty found in the *cse*-*2* mutant. Through a vessel-specific complementation approach, vasculature morphology and final stem weight were restored, leading to an even higher total glucose release per plant.

**Electronic supplementary material:**

The online version of this article (doi:10.1186/s13068-016-0551-9) contains supplementary material, which is available to authorized users.

## Background

Lignin is a polymeric substance composed of aromatic heteropolymers that provide structural integrity, stiffness, and hydrophobicity to secondary-thickened cell walls [1]. It is produced by the oxidative combinatorial coupling of mainly the hydroxycinnamyl alcohols *p*-coumaryl, coniferyl and synapyl alcohols, which differ in their degree of methoxylation. Upon their incorporation into the growing polymer, these monolignols produce *p*-hydroxyphenyl (H), guaiacyl (G), and syringyl (S) units, respectively [2], and their individual contribution to lignin composition varies significantly among taxa, tissues, and cell types [3].

Because lignification represents a highly energy-consuming process and irreversible carbon investment, the biosynthesis of lignin and other major components of secondary cell wall are tightly regulated at the transcriptional level. The transcriptional regulation of secondary cell wall deposition employs a multi-leveled hierarchical network formed by a top-level of secondary wall NAC regulators that directly activate a second level of secondary cell wall MYB master switches and, together, they activate the expression of downstream transcription factors and secondary cell wall biosynthetic genes [4]. Comprehensive studies have shown that NAC and MYB master switches regulate a number of downstream targets by binding to specific consensus elements in their promoters, which were designated ‘secondary wall NAC binding element’ (SNBE) and ‘secondary wall MYB-responsive element’ (SMRE), respectively [5, 6] Indeed, virtually all secondary wall biosynthetic genes contain at least one SNBE and one SMRE site in their promoters, including genes involved in the biosynthesis of cellulose, xylan, and lignin [4]. Despite of the recent progress in our understanding of the regulation of secondary cell wall deposition, only *MYB58*, *MYB63*, and *MYB85* have been characterized as lignin biosynthesis activators in Arabidopsis [7, 8].

The lignin biosynthetic pathway itself has been extensively studied and was thought to be fully defined over a decade ago. Therefore, the recent discovery of CAFFEOYL SHIKIMATE ESTERASE (CSE) as a new biosynthetic enzyme central to the lignin pathway was unexpected [9]. Previously, *p*-HYDROXYCINNAMOYL-CoA:SHIKIMATE/QUINATE *p*-HYDROXYCINNAMOYLTRANSFERASE (HCT) was shown to catalyze the production of *p*-coumarate esters from *p*-coumaroyl-Coenzyme A (CoA) prior to the 3-hydroxylation of the aromatic ring by *p*-COUMARATE 3-HYDROXYLASE (C3H), which then produces the corresponding caffeate esters [10, 11]. HCT was also suggested to catalyze a second reaction in the pathway, converting the resulting caffeate esters into caffeoyl-CoA, based on *in vitro* assays using caffeoyl quinate (chlorogenic acid) and CoA. The confirmation of this second reaction *in planta* by means of reverse genetics was hindered, because *HCT* downregulation also affects the biosynthesis of the upstream intermediate *p*-coumaroyl shikimate earlier in the pathway. However, this proposed role for HCT in converting caffeate esters into caffeoyl-CoA was recently challenged with the characterization of CSE, an enzyme shown to catalyze the conversion of caffeoyl shikimate into caffeate in *Arabidopsis thaliana*. The activity of CSE combined with that of 4-COUMARATE:CoA LIGASE (4CL) produces caffeoyl-CoA, a central precursor for G and S lignin synthesis, and thus bypasses the second reaction of HCT. The involvement of CSE in lignification *in planta* was further demonstrated using T-DNA insertion lines that show typical phenotypes of lignin-deficient mutants, including collapsed vessels, reduced lignin content, and lignin compositional shifts [9]. Although *CSE* orthologs have been found in other plant species, including potential bioenergy crops, such as poplar and switchgrass [9, 12], a role for CSE in lignification of plants other than Arabidopsis has only been verified in a very recent report [13]. *Medicago truncatulaCSE* loss-of-function lines due to a transposon insertion showed severe dwarfing, reduction in lignin content, and drastically increased levels of H-derived monomers. Moreover, recombinant MtCSE was able to efficiently convert caffeoyl shikimate into caffeic acid *in vitro* [13]. Although these results suggest that the CSE enzyme is critical to normal lignification in *M. truncatula*, the lack of CSE orthologs in the model grass *Brachypodium distachyon* and in maize, together with the absence of CSE activity in crude protein extracts from stems of these species [13], suggests that the enzymatic step catalyzed by CSE might not be essential for lignification in all plant species.

Although important for normal plant growth and development, lignin is a major limiting factor for the efficient processing of plant biomass for downstream applications, such as chemical pulping and biofuel production [14, 15]. Accordingly, the cellulose-to-glucose conversion efficiency of mutants or transgenic plants with reduced lignin content is usually higher when compared to that of wild-type plants [15, 16]. For example, in the *cse*-*2* loss-of-function mutant, which showed a 36 % reduction in lignin content [9], a fourfold increase in cellulose-to-glucose conversion was observed one of the highest improvements in saccharification efficiency ever reported. However, *cse*-*2* mutants exhibit a substantial developmental arrest, with their inflorescence stems being 37 % smaller and 42 % lighter at senescence than those of the wild type. Indeed, bioengineering of lignin deposition frequently results in adverse effects on plant growth and development and, consequently, on plant yield [17–20], which might outweigh the gains in fermentable sugar yield [20]. One hypothesis to explain the yield penalty is that limited lignin deposition results in collapse of xylem vessels under the negative pressure generated by transpiration, impairing water transport [18, 21]. Plants with defective lignification in interfascicular and xylary fibers but with normal lignification in vessels grow to wild-type size, despite the pendant stem phenotype [22–24], and have an improved saccharification yield due to the reduced overall lignin level [25]. Here, we evaluated whether vasculature collapse was the reason for the abnormal growth phenotype of *cse*-*2* plants by reintroducing *CSE* expression specifically into the xylem vessels, and analyzed how this approach affected the total glucose release per plant. Furthermore, we evaluated whether *CSE* expression is likely under the same transcriptional control as other lignin biosynthetic genes and analyzed the transcriptional responses of the phenylpropanoid pathway upon *CSE* loss-of-function.

## Results

### The CSE promoter is a target for secondary wall-associated transcription factors

**Fig. 1:**
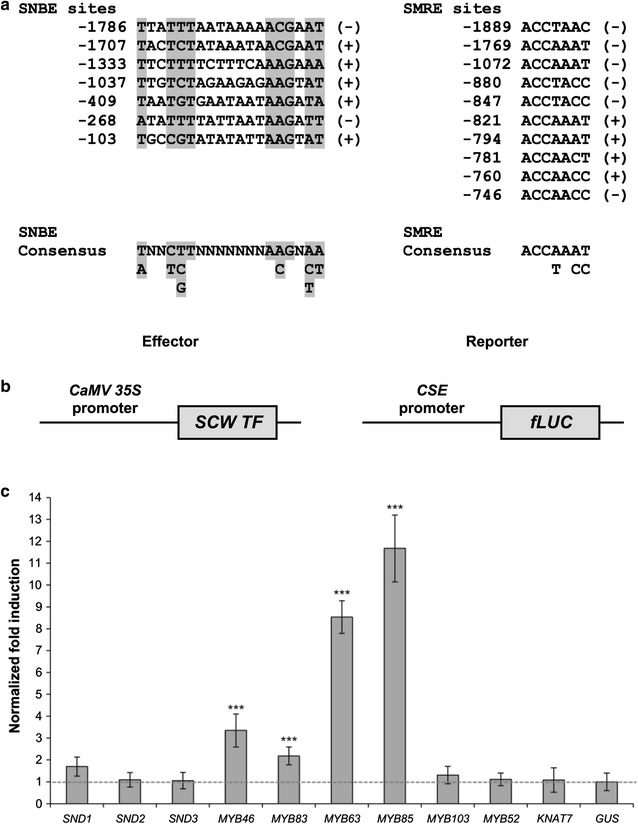
Transactivation analysis of the *CSE* promoter by secondary wall-associated transcription factors in tobacco protoplasts. **a** Putative SNBE and SMRE sequences identified from the promoter region 2-kb upstream of the start codon of *CSE*. The *numbers on the left* indicate the position relative to the translational start site, while *symbols on the right* indicate whether the sequence was identified in the forward (+) or reverse (−) strands of the DNA. The consensus nucleotides in the SNBE and SMRE sequences are shaded in *gray*. **b** Schematic representation of the effector and reporter constructs. The effector construct consists of the constitutive *CaMV* *35S* promoter driving the expression of secondary wall-associated (SCW) transcription factors, while the reporter construct consists of the *CSE* promoter driving the expression of firefly luciferase reporter gene (fLUC). **c** Transactivation analysis showing that only second-level MYB master switches (MYB46 and MYB83) and lignin-specific activators (MYB63 and MYB85) effectively activate the expression of the *proCSE*-driven fLUC reporter gene. Values are fold-changes normalized to protoplasts co-transfected with the reporter constructs and a *CaMV* *35S::GUS* control plasmid. *Error bars* indicate the standard deviation and significance was determined by Student’s *t* test (***P < 0.001; *n* = 8)

Because *CSE* is a newly discovered gene of the lignin biosynthesis pathway, we investigated whether the *CSE* promoter also contained the SNBE and SMRE sites and, consequently, whether it could be activated by secondary wall-associated transcription factors. The forward and reverse strands of a region 2-kb upstream of the start codon were examined for the consensus sequence of SNBE [(T/A)NN(C/T)(T/C/G)TNNNNNNNA(A/C)GN(A/C/T)(A/T)] and of SMRE [ACC(A/T)A(A/C)(T/C)] [5, 6]. The *CSE* promoter contained a total of seven SNBE sites, five in the forward and two in the reverse strand, and ten SMRE sites, four in the forward and six in the reverse strand (Fig. 1a). Since the presence of these binding elements does not necessarily imply that they are functional elements [6], we ligated the 2-kb promoter region of *CSE* upstream of the firefly luciferase reporter gene and tested the activity of previously characterized secondary wall-associated transcription factors in transactivation assays in tobacco protoplasts (Fig. 1b, c). We found that MYB63 and MYB85, transcriptional regulators that specifically activate lignin biosynthesis, and significantly and strongly induced the expression of the reporter gene. The MYB46 and MYB83, which are functionally redundant activators of secondary cell wall biosynthetic genes for cellulose, xylan and lignin, were also able to activate the *CSE* promoter, albeit to a lesser extent (Fig. 1c). Interestingly, no significant induction of luciferase expression was observed for the tested NACs, neither for MYB52, MYB103 and KNAT7. Within the limits of the experimental system used, these results suggest that *CSE* is not a target of NAC master switches at the top level of the transcriptional network controlling secondary cell wall deposition in Arabidopsis, but rather that its expression is regulated by the second-level MYB master switches (e.g., MYB46 and MYB83) and, especially, by lignin-specific activators, such as MYB63 and MYB85.

### Lignin biosynthetic genes upstream of *CSE* are downregulated in the *cse-2* mutant

**Fig. 2:**
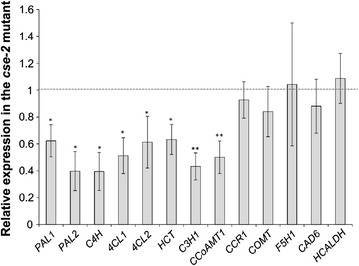
Expression analysis of lignin biosynthetic genes in inflorescence stems of the *cse*-*2* mutant and the wild type as determined via RT-qPCR. The relative expression of each tested gene was normalized to that of the wild type. *Error bars* indicate the standard deviation. Differences in gene expression were assessed with Student’s *t* test (*0.05 > *P* > 0.01; **0.01 > *P* > 0.001; *n* = 4)

In a previous work, we investigated the system-wide responses of perturbations in consecutive steps of the lignin biosynthetic pathway in a set of Arabidopsis single mutants [26]. This approach revealed a transcriptional feedback of phenylpropanoid genes upon blocking particular steps in monolignol biosynthesis [9], leading to the hypothesis that reduced lignin content triggers upregulation of the early steps in the biosynthesis of monolignols. To investigate whether this hypothesis holds also true for the low-lignin *cse*-*2* mutant, we evaluated the transcript levels of lignin biosynthetic genes in inflorescence stems of *cse*-*2* via quantitative RT-PCR (Fig. 2). However, in contrast to other mutants with reduced lignin levels, the *cse*-*2* mutant downregulated all genes upstream in the pathway, while no significant differences were observed for genes downstream of *CSE*, with the exception of *CCoAOMT1*, which was also downregulated. The expression of *HYDROXYCINNAMALDEHYDE DEHYDROGENASE* (*HCALDH*), an enzyme required for the biosynthesis of ferulic and sinapic acid [27], was not affected by CSE deficiency either. These data suggest that the lower lignin content of the *cse*-*2* mutant might result from a combination of blocking the biosynthetic pathway and reducing the expression levels of genes that supply monolignol precursors.

### Vessel-specific complementation of *cse-2* further improves saccharification yield per plant

**Fig. 3:**
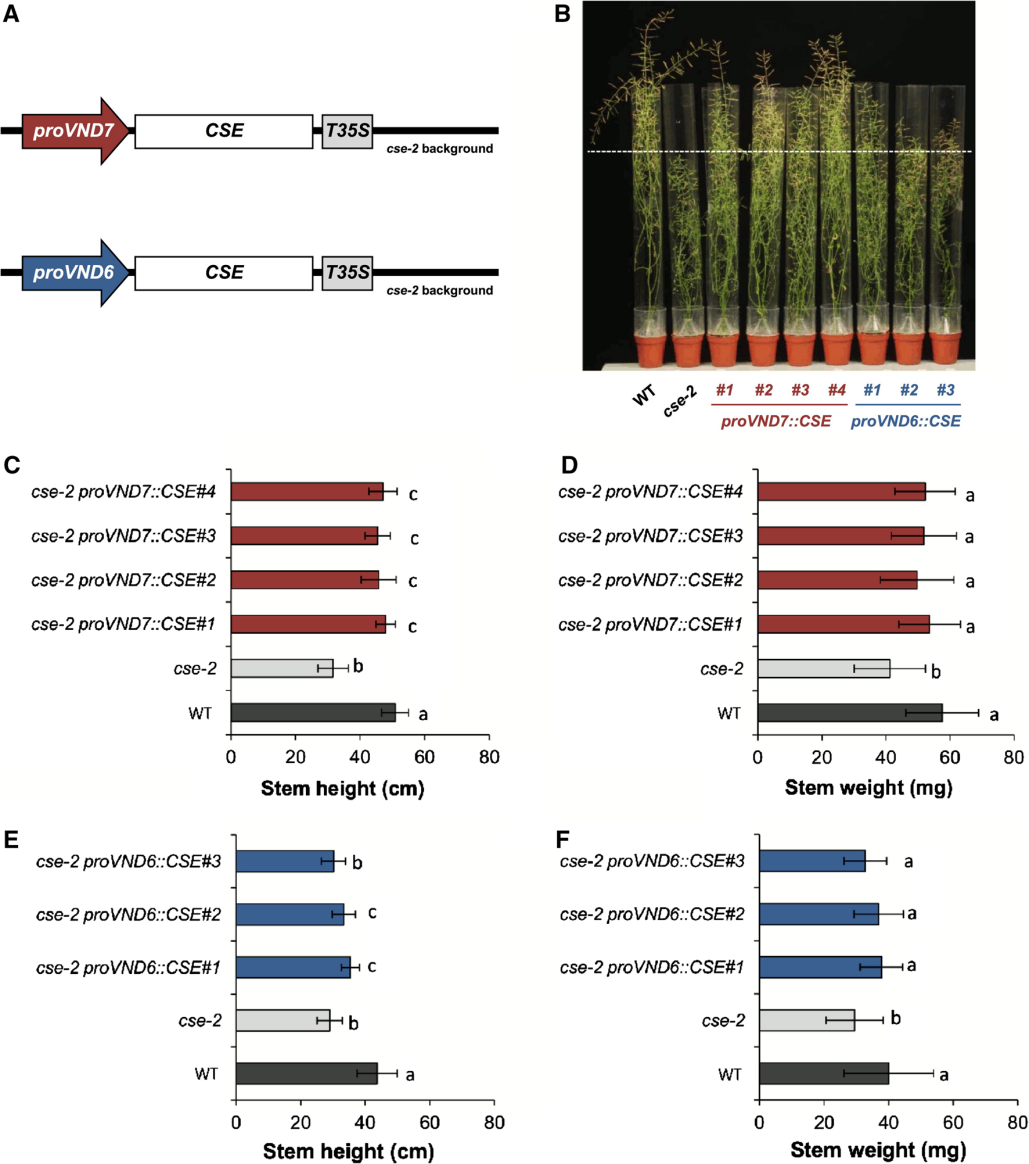
Vessel-specific expression of *CSE* in the *cse*-*2* mutant background partially restored plant growth. **A** Schematic illustration of the expression cassette used for the vessel-specific complementation of *cse*-*2*. *VND* promoters are indicated as *arrows*, while the *CSE* coding sequence and the *35S* terminator (*T35S*) are represented as *rectangles*. *Black line* represents the *cse*-*2* genetic background. **B** Phenotype of fully-grown plants after 8 weeks of short-day growth conditions and transferred to long-day growth conditions for an additional 5 weeks. Final height (**C**, **E**) and weight (**D**, **F**) of the main inflorescence stem at senescence for the *cse*-*2 proVND::CSE* lines, the *cse*-*2* mutant, and the wild type. Significant differences between the mean values are based on a nested ANOVA model. *Error bars* indicate the standard deviation

The potential of *CSE* as a general target for reducing cell wall recalcitrance has already been proven, because the improvement in saccharification efficiency of the Arabidopsis *cse*-*2* mutant is among the highest reported so far [9, 15]. However, as is the case with many lignin mutants, CSE deficiency results in a significant loss of total biomass yield (~40 %). Because the reduced lignin content in *cse*-*2* also results in the so-called irregular xylem (*irx*) phenotype [9], we hypothesized that the main reason for the yield penalty of the mutant is the presence of collapsed vessels and that restoring lignin deposition exclusively in vessels would also restore plant growth. To this end, we used the vessel-specific promoters *VASCULAR*-*RELATED NAC DOMAIN 6* (*VND6*) and *VND7* to drive the expression of *CSE* in the *cse*-*2* mutant background (Fig. 3A). VND6 and VND7 are master switches of the NAC family, which control the activation of secondary cell wall biosynthesis in metaxylem and protoxylem vessels, respectively [28, 29], and their tissue-specific expression patterns have been already exploited in similar biotechnological strategies to restore secondary cell wall biosynthesis specifically in vessels [25, 30]. A total of three and four independent lines harboring the *cse*-*2* mutation and expressing either the *proVND6::CSE* or the *proVND7::CSE* constructs, respectively, were selected for further analyses (Fig. 3B; Additional file 1).

To first evaluate whether the *proVND::CSE* constructs successfully restored plant growth, the *cse*-*2proVND::CSE* lines were grown alongside the *cse*-*2* mutant and the corresponding wild type under short-day conditions for 8 weeks and subsequently moved to long-day conditions. These growth conditions allow the development of a large, single inflorescence stem to maximize secondary cell wall thickening [26]. The final dry weights of the main stem of all four *cse*-*2 proVND7::CSE* lines were not significantly different from that of the wild type while significantly heavier (21–30 %) than that of the *cse*-*2* mutant (Fig. 3D). However, the final height was only partially restored in these lines, with inflorescence stems still being between 6 and 11 % shorter than those of the wild type (Fig. 3C). Nevertheless, these plants were still significantly taller (44–51 %) than *cse*-*2* plants, whose inflorescence stems were 38 % shorter than those of the wild type. Similar results were obtained for two out of three *cse*-*2 proVND6::CSE* lines, with fully complemented weight but partially restored height (inflorescence stems of *cse*-*2 proVND6::CSE#1* and *#2* being 19 and 23 % shorter than wild type and 22 and 15 % taller than *cse*-*2* plants, respectively; Fig. 3E, F).

**Fig. 4:**
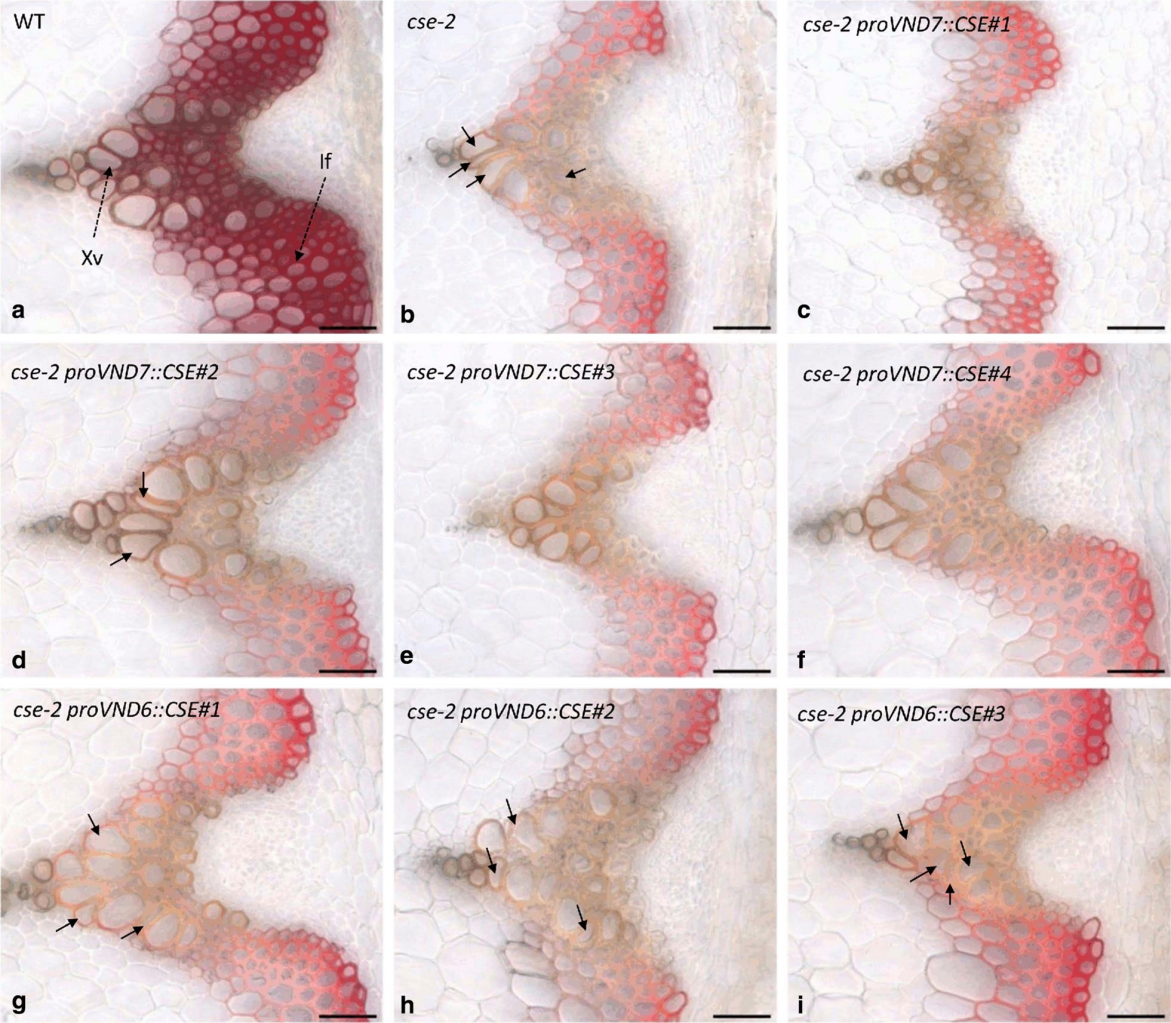
Bright-field microscopy of stem cross sections from the wild type, *cse-2* mutant and the *cse-2 proVND::CSE* lines after Mäule staining. Compared to the wild type (**a**), *cse-2* mutants (**b**) showed reduced staining, indicative of reduced lignin deposition, and irregularly shaped vessels (*solid arrows*). An intermediate staining intensity of xylem vessels was observed in *cse-2 proVND7::CSE* lines (**c**–**f**), with normal shaped vessels, except eventually for *cse-2 proVND7::CSE#2*. Vasculature morphology of *cse-2 proVND6::CSE* lines (**g**–**i**) resembled that of the* cse-2* mutant. *If*: Interfascicular fibers; *Xv*: xylem vessels; *Bar* 50 µm.

Next, we analyzed the effect of the *proVND6/proVND7::CSE* constructs on vascular tissue morphology and on lignin deposition in stem cross sections. Histochemical analysis using Mäule reagent, which stains G lignin brown and S lignin pink, showed that wild-type xylem vessels are large, round-shaped cells enriched in G-type lignin, while wild-type interfascicular fibers are small, heavily lignified cells enriched in S-type lignin (Fig. 4a). *cse*-*2* mutants showed reduced lignin deposition in both cell types and developed irregularly shaped vessels, which were often smaller when compared to those of the wild type (Fig. 4b). Mäule staining further revealed a modest increase in staining intensity of xylem vessels in *cse*-*2 proVND7::CSE* lines, while lignin was still reduced in the interfascicular fibers (Fig. 4c–f). Conversely, staining intensity of xylem vessels in *cse*-*2 proVND6::CSE* lines resembled that of the *cse*-*2* mutant (Fig. 4g–i). To evaluate the effectiveness of the complementation approach in restoring vascular morphology, a semi-quantitative assessment of the *irx* phenotype was performed by visually scoring the number of collapsed vessels per vascular bundle for each genotype (Additional file 2). While the number of collapsed vessels of the *cse*-*2 proVND7::CSE* lines was not statistically different from that of the wild type (Additional file 2), a significant number of collapsed vessels were still observed in the *cse*-*2 proVND6::CSE* lines, although in lower frequency when compared to *cse*-*2* mutant (Fig. 4g–i; Additional file 2). These results show that vascular morphology was nearly completely restored when the *VND7* promoter was used to drive *CSE* expression in the *cse*-*2* background, but only partially when the *VND6* promoter was used. Therefore, all subsequent cell wall analyses and saccharification assays were performed only on plant material derived from *cse*-*2 proVND7::CSE* lines.

To evaluate the impact of the vessel-specific complementation on lignin deposition in more detail, crude cell wall residue (CWR) of fully senesced stems was prepared through sequential extraction and analyzed for lignin content and composition. The CWR was about 80 % of the dry weight for all lines used in this study (Table 1). Total lignin amounts of all *cse*-*2 proVND7::CSE* lines, estimated by the acetyl bromide (AcBr) method, were significantly lower than that of the wild type (Table 1). Interestingly, only two out of the four *cse*-*2 proVND7::CSE* lines (#3 and #4) showed a slight but significant increase in total lignin content compared to the *cse*-*2* mutant. A minor effect on the overall lignin content in these lines is consistent with the recovery of lignin deposition specifically in vessels. Subsequently, lignin monomeric composition was analyzed by thioacidolysis, a procedure that quantifies lignin monomers linked by β-O-4-ether bonds. As previously reported, the *cse*-*2* mutant showed an unconventional lignin composition, with remarkably high amounts of H units and reduced levels of both G and S units compared to the wild type based on CWR (Table 1). Consequently, changes in the H:G:S ratio were observed from about 1:62:37 in the wild type, to about 50:25:25 in the *cse*-*2* mutant (Table 1). The H:G:S ratio of three out of four *cse*-*2 proVND7::CSE* lines (#1, #2, and #3) did not differ significantly from those of *cse*-*2*, while the fourth line, *cse*-*2 proVND7::CSE#4*, had a slightly lower relative H and higher relative S frequency (H:G:S of 41:28:31) as compared to the *cse*-*2* mutant. The degree of lignin condensation is inversely correlated with the sum of H, G, and S units released via thioacidolysis. Accordingly, the 81 % reduction in H + G + S release in *cse*-*2* as compared to wild type, indicated a higher degree of lignin condensation in the *cse*-*2* mutant (Table 1). The H + G + S release from the *cse*-*2 proVND7::CSE* lines did not significantly differ from that of the *cse*-*2* mutant when calculations were based on the amount of AcBr lignin. However, a significant increase of 73 % in H + G + S release was found for *cse*-*2 proVND7::CSE#2* compared to *cse*-*2* mutant when calculations were based on the amount of CWR.

**Table 1:** Cell wall characterization of *cse*-*2 proVND7* lines

	WT	*cse-2*	*cse-2 proVND7::CSE#1*	*cse-2 proVND7::CSE#2*	*cse-2 proVND7::CSE#3*	*cse-2 proVND7::CSE#4*
CWR/DW (%)	80.5 (1.3)a	79.5 (2.1)a	80.2 (1.4)a	79.8 (1.3)a	79.8 (2.4)a	81.6 (3.0)a
AcBr lignin/CWR (%)	13.2 (1.4)a	6.5 (0.4)b	7.1 (0.3)b, c	7.3 (0.7)b, c	7.4 (0.3)c	7.6 (0.5)c
Cellulose/CWR (%)	37.64 (0.09)a	38.89 (0.04)a	47.80 (0.13)a	41.00 (0.02)a	42.25 (0.03)a	39.08 (0.05)a
Cellulose/DW (%)	31.37 (0.07)a	31.59 (0.04)a	39.04 (0.1)a	33.42 (0.02)a	34.41 (0.03)a	31.90 (0.03)a
H units/CWR (µmol/g)	3.4 (1.4)a	43.9 (14.1)b	61.6 (9.9)b, c	65.9 (5.8)c	60.7 (8.1)b, c	54.5 (14.7)b, c
G units/CWR (µmol/g)	291.7 (152.8)a	21.3 (4.1)b	38.7 (6.6)c	41.4 (6.9)c	33.3 (3.3)b, c	39.3 (17.0)b, c
S units/CWR (µmol/g)	175.2 (94.5)a	22.2 (6.2)b	39.6 (7.8)b, c	43.9 (7.8)c	35.4 (5.2)b, c	44.4 (22.9)b, c
H + G + S units/CWR (µmol/g)	470.4 (248.2)a	87.4 (22.8)b	140.0 (22.8)b, c	151.2 (20.2)c	129.5 (12.4)b, c	138.2 (49.7)b, c
H units/AcBr lignin(µmol/g)	26 (10)a	672 (217)b	867 (123)b	913 (139)b	815 (121)b	725 (202)b
G units/AcBr lignin (µmol/g)	2210 (1071)a	328 (74)b	547 (96)b, c	576 (140)c	447 (58)b, c	512 (191)b, c
S units/AcBr lignin (µmol/g)	1323 (654)a	342 (106)b	559 (109)b	610 (142)b	476 (87)b	575 (257)b
H + G + S units/AcBr lignin (µmol/g)	3559 (1730)a	1342 (370)b	1974 (308)b	2099 (419)b	1739 (221)b	1813 (558)b
%H	0.8 (0.1)a	49.6 (5.2)b	44.1 (2.8)b	43.9 (2.3)b	46.9 (4.1)b	41.1 (8.7)b
%G	62.1 (1.7)a	25.0 (3.7)b	27.7 (1.4)b	27.3 (1.4)b	25.9 (2.9)b	27.8 (2.6)b
%S	37.1 (1.6)a	25.4 (2.5)b	28.2 (2.1)b,c	28.9 (1.5)b, c	27.3 (2.0)b, c	31.0 (6.2)c
S/G	0.6 (0.04)a	1.03 (0.15)b	1.02 (0.08)b	1.06 (0.07)b	1.06 (0.11)b	1.11 (0.13)b

Lignin amount was expressed as percentage of CWR, while the amount of each subunit was expressed in μmol g^−1^ CWR and in μmol g^−1^ AcBr lignin. The relative proportions of the different lignin units were calculated based on the total thioacidolysis yield. Cellulose content was expressed as percentage of CWR. Numbers between brackets indicate the standard deviation. One-way ANOVA and the Tukey HSD post hoc tests were performed to reveal significant (*P* < 0.05) differences between the various lines, which are indicated by different letters

**Fig. 5:**
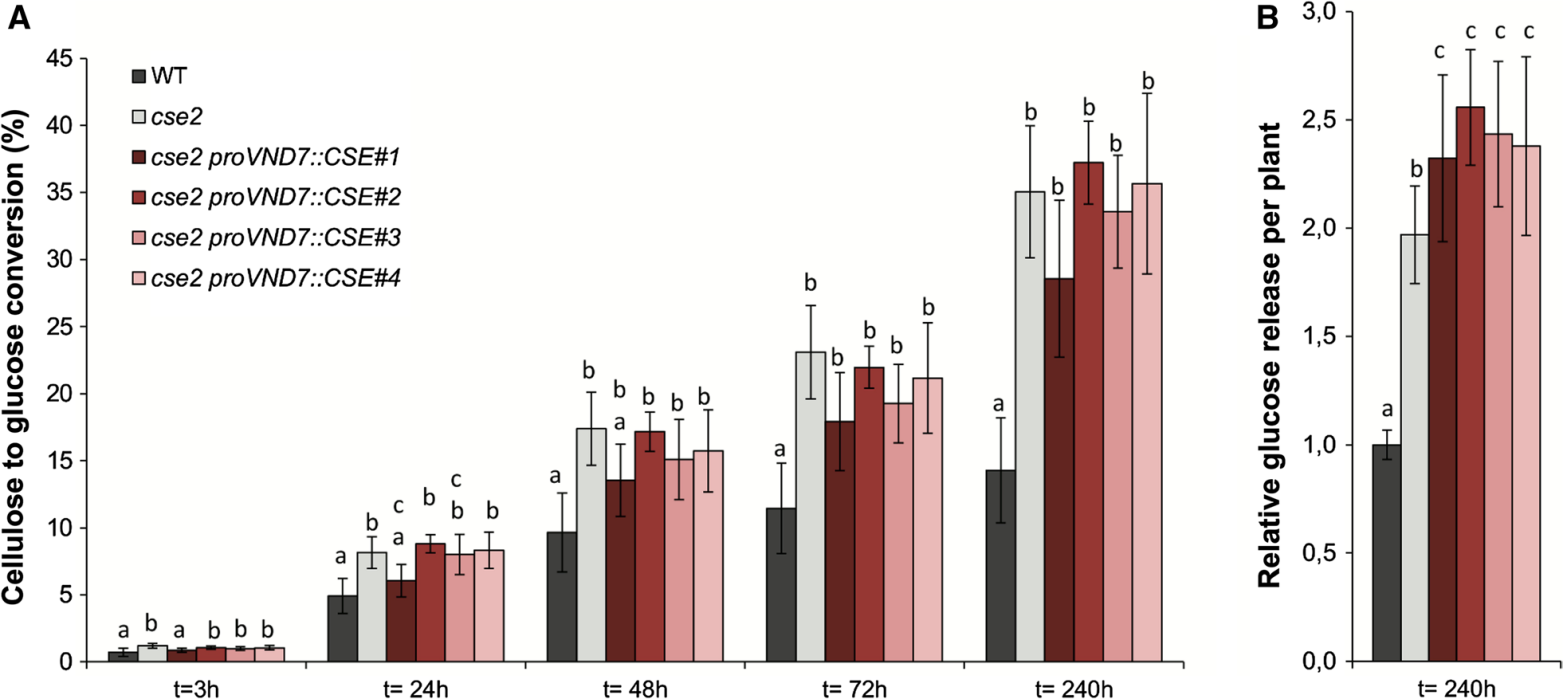
Vessel-specific expression of *CSE* in the *cse*-*2* mutant background further improves saccharification efficiency. **A** Cellulose-to-glucose conversion during saccharification of the senesced stems of the wild type, *cse*-*2* mutant and the *cse*-*2 proVND7::CSE* lines without pretreatment. The cellulose-to-glucose conversion was measured after 3, 24, 48, 72, and 240 h. **B** Relative glucose release per plant after 240 h of saccharification without pretreatment, normalized to the values of the wild type. *Error bars* indicate the standard deviation. Significant differences were assessed with one-way ANOVA, after Bartlett’s test, and the Tukey HSD was performed as post hoc test. *Different letters* represent significant differences (*P* < 0.05)

Because lignin content and composition of the *cse*-*2 proVND7::CSE* lines remained similar to those of the *cse*-*2* mutant, we anticipated that their biomass would also remain easier to saccharify when compared to the wild type. First, the cellulose content of CWR prepared from senesced stem material was measured using the phenol–sulfuric acid method. No differences were found in cellulose levels among all the analyzed genotypes, regardless whether the values were expressed relative to CWR or to the dry weight (Table 1). Next, the saccharification yield, measured as the amount of glucose (Glc) released by the enzymatic degradation of cellulose, was determined according to a previously established protocol for small biomass samples [15, 31]. Senesced stems of the wild-type, *cse*-*2* mutant and the *cse*-*2 proVND7::CSE* lines were cut into 2-mm pieces and subjected to saccharification without pretreatment, with Glc release measured after 3, 24, 48, 72, and 240 h. The results showed that the cellulose-to-glucose conversion was significantly faster and higher for all four *cse*-*2 proVND7::CSE* lines than for wild type (Fig. 5A). For three of the four *cse*-*2 proVND7::CSE* lines (#2, #3, and #4), the cellulose-to-glucose conversion did not significantly differ from that of the *cse*-*2* mutant, while for *cse*-*2 proVND7::CSE#1*, the yield was slightly lower. Because the expression of *proVND7::CSE* into the *cse*-*2* background restored the final weight of the dry stems of fully senesced plants, we further compared the genotypes for their saccharification yield on a plant basis (i.e., total Glc release per plant). Even considering the growth penalty, the *cse*-*2* mutant released 97 % more Glc than the wild type on a plant basis (Fig. 5B). As anticipated, vessel-specific complementation of the *cse*-*2* mutant further improved the Glc release, with yields being 25–36 % higher when compared to that of the *cse*-*2* mutant and 134–154 % higher when compared to that of the wild type, with *cse*-*2 proVND7::CSE#2* as the best saccharifying of all four lines (Fig. 5B).

## Discussion

The expression of genes involved in secondary cell wall deposition is highly coordinated, both temporally and spatially, through the action of a complex regulatory network of developmental master switches, especially NAC and MYB transcription factors [32]. We identified several NAC and MYB binding elements in the *CSE* promoter region, but only members of the MYB family were able to activate this promoter in transactivation analyses. These results are in line with the observation that although all lignin genes in Arabidopsis contain at least one SNBE site in their promoters, they appear not to be direct targets of VND7 and SND1; the NAC master switches that activate secondary wall biosynthesis in vessels and fibers, respectively [6, 33]. In contrast, the expression of many lignin biosynthetic genes is directly activated by the two functionally redundant MYB transcription factors, MYB46 and MYB83 [5], which are second-level master switches, and by MYB58 and MYB63 [8], which were shown to be specific transcriptional activators of lignin biosynthesis. Moreover, by re-analyzing previously published transcriptomic datasets, we found that *CSE* was among the direct target genes of MYB46, with a similar fold-induction to that of other lignin biosynthetic genes [5], but not among the direct targets of the secondary wall master switches SND1 [6] and VND7 [33], corroborating the results from our protoplast transactivation assay. Altogether, these findings suggest that *CSE* expression is regulated by MYB transcription factors positioned at different levels in the regulatory network controlling secondary cell wall deposition in Arabidopsis, similar to other lignin biosynthetic genes. Noteworthy, as transient expression assays in plant protoplasts provide only preliminary information on the function of transcription factors, based on their transactivation properties, further studies are needed to unambiguously demonstrate how the expression of *CSE* and other lignin genes is regulated.

Altering the expression of genes in the lignin biosynthetic pathway has far-reaching consequences on the transcriptome and metabolome, resulting in shifts in both primary metabolism and secondary metabolism [26, 34–38]. We have previously shown that mutants with lower lignin content (i.e., *c4h*, *4cl1*, *ccoaomt1*, and *ccr1*) generally upregulate genes involved in the production of monolignols, whereas mutants with compositional shifts (i.e., *f5h1* and *comt*) rather downregulate genes of this pathway [26]. The suggested causal reason was the accumulation of soluble G-type compounds (e.g., coniferyl alcohol, coniferin, or G-type oligomers), which may work as regulators of the phenylpropanoid pathway. This hypothesis was supported by an inverse correlation between the levels of G-type metabolites and the transcript levels of the shikimate and phenylpropanoid pathway genes in Arabidopsis lignin mutants [26], in CCR-deficient tobacco and poplar [35, 39], and in *F5H1* overexpressing (*proC4H:F5H1*) and *comtproC4H:F5H1* Arabidopsis lines in which G-type compounds are replaced by S- and 5-hydroxyguaiacyl-type compounds, respectively [40]. Surprisingly, the *cse*-*2* mutant, with both reduced lignin content and compositional shift, exhibits transcriptional repression of genes upstream of *CSE*, while downstream genes were mainly unaffected. Possibly, CSE disruption leads to the accumulation of specific pathway intermediates or derivatives that trigger a transcriptional repression of phenylpropanoid genes even when the levels of lignin, and hence G-type metabolites, are low. Moreover, the downregulation of many phenylpropanoid biosynthesis genes in the *cse*-*2* mutant background should be considered when specific promoters are chosen for genetic engineering approaches. For example, the *C4H* promoter has been used as a strong, vascular-specific promoter to drive the expression of genes in lignifying tissues, and frequently outperforms the constitutive, non-tissue-specific *CaMV35S* promoter [40–44]. However, due to the significant downregulation of *C4H* in the *cse*-*2* background, its promoter would not be an obvious choice to drive gene expression in a *cse* background. Other secondary cell wall-specific promoters should be considered, such as the Arabidopsis *CesA7* promoter, which has already been demonstrated to strongly drive the expression of a target gene specifically in cells with thickened cell walls in Arabidopsis [24, 45], and whose expression is not correlated with the expression of lignin genes in a series of lignin mutants [26].

Engineering of lignin in lignocellulosic feedstock has been widely recognized as an effective strategy to improve biomass conversion efficiency. The most straightforward and widely used approach for lignin bioengineering has been the reduction of its content in plant biomass by the modification of the expression of biosynthetic enzymes [14, 15, 46–48]. However, drastic, non-selective reductions in lignin deposition often cause dwarfism or detrimental developmental effects, highlighting the limitations of using constitutive silencing approaches. Accordingly, the overall lignin content is reduced by 36 % upon knocking out *CSE* in Arabidopsis, but the plant growth and development are substantially affected, with the mutant inflorescence stems being 37 % smaller and 42 % lighter at senescence when compared to the wild type [9]. Recently, more sophisticated approaches have been added to the lignin bioengineering toolbox, allowing a more precise modification of lignin with reduced or no detrimental effects at all on plant development [reviewed in 49]. For instance, employing tissue-specific promoters may allow altering lignin deposition in tissues crucial for biomass conversion (e.g., fibers) without perturbing lignin biosynthesis in tissues important for the plant physiology (e.g., vessels). Such an approach, in which the yield penalty of the Arabidopsis *c4h* mutant was (partly) overcome by restoring lignin biosynthesis in vessel cells, thus rescuing the *irx* phenotype, has been recently reported [25]. Because the *cse*-*2* mutant also shows the *irx* phenotype, we hypothesized that a vessel-specific complementation approach might successfully restore plant growth and, consequently, further improve the saccharification yield.

In general, expression of *CSE* under the control of the *VND6* and *VND7* promoters in the *cse*-*2* background resulted in improved growth for most of the lines when compared to the *cse*-*2* mutant. Interestingly, the *VND7* promoter worked significantly better than the *VND6* promoter, with all the *cse*-*2 proVND7::CSE* lines showing complete recovery of the final stem weight and partial recovery of the final stem height. Moreover, collapsed vessels were largely absent in *proVND7::CSE* lines, but still frequently observed in stem cross sections of *cse*-*2 proVND6::CSE* lines. Therefore, further cell wall analyses and saccharification assays were only performed with *cse*-*2 proVDN7::CSE* lines. Our results contrast with a previous study, where the *VND6* promoter was used to drive the expression of *C4H* in an Arabidopsis *c4h* mutant background [25]: microscopy of the *c4h proVND6::C4H* inflorescence stem sections showed an enhanced lignification in vessels and to a lesser extent in the interfascicular fiber region [25]. On the other hand, our findings are in agreement with the results from the vessel-specific complementation of the growth phenotype of xylan deficient *irx* mutants in Arabidopsis [30]. In the latter study, the complementation of the biomass yield was better when the *VND7* promoter was used than when the *VND6* promoter was used [30]. The different outcomes of using the *VDN6* or *VND7* promoter might be due to the distinct expression patterns of these promoters. When the *VND* gene family was first characterized in Arabidopsis, the expression of *VND6* and *VND7* was shown to be restricted to metaxylem and protoxylem vessels, respectively [28] in roots of 7-day-old seedlings. Subsequent work has demonstrated that the expression of *VND6* is limited to the inner metaxylem vessels of the root elongation/differentiation zones, while *VND7* is expressed in both protoxylem and metaxylem vessels throughout the seedlings [29]. This broader expression pattern may explain the better performance of the *VND7* promoter in our complementation approach and the one of Petersen and colleagues [30]. However, we cannot exclude that the better performance observed for the *VND7* promoter is due to position effects, and that *proVND6* would be as efficient if more lines would have been analyzed. Noteworthy, a recent study has found different expression patterns for the *VND1* to *VND5* genes when compared to the original work of Kubo et al. [28], and this discrepancy was explained by the use of translational reporter constructs with the full length gene sequence instead of transcriptional reporter constructs, since regulatory cis-elements for tissue-specific expression might be intragenically located [50]. Thus, we cannot exclude the possibility that the capacity of vessel-specific complementation using *VND* promoters is affected by the lack of all cis-elements required for a proper expression in vessels.

By reintroducing the CSE function specifically in vessels, it was possible to recover the final stem weight and complement the *irx* phenotype of the *cse*-*2* mutant, while all the lines still had reduced the overall lignin levels that were either intermediate between the mutant and the wild type or as low as that of the *cse*-*2* mutant. That lignin levels had remained low in the vessel-complemented lines is not surprising, because it is known that the lignification of vessels is (partially) a non-cell autonomous process, requiring neighboring cells to produce monolignols to achieve full lignification after the rapid programmed cell death of the vessels [24, 51, 52]. In the *cse*-*2 proVND7::CSE* lines, monolignol biosynthetic activity of neighboring cells is still compromised, while the developing vessels, whose ability to deposit lignin was rescued by *proVND7::CSE* expression, undergo rapid programmed cell death, resulting in only partial lignification. Interestingly, Petersen et al. [30] found similar results for the vessel-specific complementation of xylan biosynthesis in Arabidopsis *irx* mutants; the growth phenotype of the plants was restored, while the amount of xylan in the walls of xylem vessels had not fully restored. Noteworthy, Yang et al. [25] observed a significant increase in lignin levels in their *c4h proVND6::C4H* rescued lines, which were intermediate between WT and the *c4h* mutant, suggesting a significant activity of the *VND6* promoter in xylem vessels of Arabidopsis inflorescence stems. Thus, our results show that it is possible to rescue the growth phenotype of *CSE*-deficient plants by restoring *CSE* expression specifically in xylem vessels, supporting earlier findings that vascular integrity is important to avoid the yield penalty associated with reductions in lignin or other secondary wall components [25, 30].

Similar to many lignin-deficient mutants in Arabidopsis, the cell walls of *cse*-*2* exhibit a higher saccharification efficiency compared to the wild type, but total biomass is significantly reduced. By restoring the vascular integrity of *cse*-*2*, and consequently its biomass parameters, we aimed to overcome the losses in fermentable sugar release caused by the yield penalty. Accordingly, the expression of *proVND7::CSE* in the *cse*-*2* background further improved glucose release per plant without pretreatment, leading to an increase of up to 36 % compared to the mutant and up to 154 % compared to the wild type. This result is in accordance with the previous studies in which approaches aiming to reduce the overall lignin contents, while maintaining intact functional vessels, improved biomass digestibility in Arabidopsis [25, 30]. Moreover, since extraxylary fiber cells contribute to a substantial part of total plant biomass in potential bioenergy crops, such as grasses and woody plants, similar strategies in crops open new perspectives for the development of the next-generation bioenergy feedstocks.

Noteworthy, the growth phenotype observed in many lignin-deficient mutants is apparently not always related to vasculature integrity, and other mechanisms have been suggested to be causative to the yield penalty. Recently, it has been shown that the levels of the stress hormone salicylic acid (SA) are inversely proportional to lignin levels in a series of transgenic alfalfa plants independently perturbed in different steps of lignin biosynthesis [53]. It was subsequently shown that the growth phenotype of Arabidopsis RNAi-HCT lines was significantly alleviated when SA levels were reduced by either genetically blocking its formation or causing its removal, suggesting that SA mediates events that orchestrate the stunted growth of lignin down-regulated plants [54]. However, although the Arabidopsis *reduced epidermal fluorescence8* (*ref8*-*1*) mutant, which has reduced C3H activity, also accumulates higher levels of SA than the wild type, the depletion of this compound in the *ref8*-*1* background did not alleviate its growth defects, indicating that the hyperaccumulation of SA is not the cause of dwarfism in this mutant [55]. Rather, the *ref8*-*1* growth phenotype is at least partially dependent on the transcriptional co-regulatory complex, Mediator [56]. The disruption of the Mediator complex subunits *MED5a* and *MED5b* led to an almost complete rescue of the *ref8*-*1* mutant growth phenotype, lignin deficiency, and changes in the expression of phenylpropanoid genes, implicating the Mediator complex in an active transcriptional process responsible for dwarfing and inhibition of lignin biosynthesis [56]. However, the stunted growth was not completely restored, especially when Mediator was disrupted in the *ref8*-*2* null mutant background, which suggests that still other mechanisms contribute to the growth phenotype observed in these mutants. Interestingly, almost completely rescued *med5a/5b ref8*-*1* mutants also accumulated higher levels of salicylic acid than *ref8*-*1* plants, confirming that SA is unlikely to be responsible for *ref8*-*1* growth defects [56]. These results, together with the fact that the phenotypes of many mutants affected in different steps of the phenylpropanoid pathway are quite diverse, imply that the dwarfing in lignin mutants is caused by different mechanisms and depends on the particular step of the pathway that is blocked. Further in line with this hypothesis is that the stunted growth of the *c4h* mutant was not rescued by the disruption of the Mediator [56], while it was successfully restored by the vessel-specific restoration of lignin biosynthesis [25]. These findings highlight the importance of understanding the cause of the dwarfism in lignin mutants to allow the development of optimized strategies to reduce plant biomass recalcitrance without affecting yield.

## Conclusions

In this study, we further characterized the involvement of the Arabidopsis *CSE* gene in lignin biosynthesis in Arabidopsis. Transactivation analysis demonstrated that only second-level MYB master switches (MYB46 and MYB83) and lignin-specific activators (MYB63 and MYB85) effectively activate the *CSE* promoter, while no transactivation activities were observed for NAC master switches and other downstream transcription factors. We found evidence for a transcriptional feedback mechanism on the phenylpropanoid pathway in *cse*-*2* plants, with transcriptional repression of genes upstream of *CSE*, while downstream genes were mainly unaffected. Therefore, a combination of blocking the biosynthetic pathway and reducing the expression levels of genes that supply monolignol precursors might explain the lower lignin content of *cse*-*2*. In addition, we found that vasculature collapse is likely the underlying mechanism causing the yield penalty in *cse*-*2* mutants. Expression of *CSE* under the control of the *VND7* promoter in the *cse*-*2* background restored the vasculature integrity resulting in improved growth while maintaining reduced overall lignin content. Importantly, by restoring the vascular integrity and biomass of *cse*-*2*, we further improved glucose release per plant in up to 36 % compared to the mutant and up to 154 % compared to the wild type. Our results confirm that restoring/maintaining vasculature integrity in plants engineered to deposit lower overall amounts of lignin is a promising strategy to reduce biomass recalcitrance without a severe yield penalty.

## Methods

### Plant material

All described *A. thaliana* lines are in the Columbia-0 ecotype. A previously described knockout mutant *cse*-*2* (SALK_023077), with an insertion in the second exon of *CSE* (At1g52760), was used in this study [9]. For vessel-specific complementation, the *cse*-*2* mutant was transformed with *proVND6::CSE* and *proVND7::CSE*. To this end, the coding sequence of *CSE* was PCR-amplified from cDNA obtained from wild-type stems and cloned into the pDONR221 vector using BP Clonase (Invitrogen) (Additional file 3). In addition, a region of 1 kb upstream of the *VND6* open reading frame [28] and 2 kb upstream of the *VND7* open reading frame were PCR-amplified from wild-type DNA using primers containing the restriction sites for BamHI (forward primer) and XhoI (reverse primer) (Additional file 3). The PCR products were cloned into the Gateway pEN-R4L1 vector using T4 DNA Ligase (Invitrogen) to generate pEN-L4-proVND6-R1 and pEN-L4-proVND7-R1, respectively. Sequence identities were confirmed by sequencing. Subsequently, the two building blocks pENTR-proVND6/VND7 and pENTR-CSE were introduced into the destination vector pK7m24GW-FAST via Multisite LR Clonase Plus (Invitrogen), which resulted in *proVND6::CSE* and *proVND7::CSE* expression clones. All the recombinant plasmids were introduced into *Agrobacterium tumefaciens* strain C58C1 PMP90 by electroporation. After plant transformation using the floral dip method, the identification of transformed seeds was based on seed fluorescence [57].

### Protoplast transactivation assay

The *CSE* promoter entry clone described above was subcloned via LR Clonase (Invitrogen) into the destination vector pm42GW7 to generate the reporter vector driving the expression of the firefly luciferase gene [58]. The ORF clones of the secondary wall-associated transcription factors were obtained from the Arabidopsis Biological Resource Center (stock numbers U16973, G85333, U86850, DQ446863, DQ056658, and G85140 for *MYB46*, *MYB83*, *MYB63*, *MYB85*, *SND2*, and *KNAT7*, respectively) or cloned from cDNA into pDONR221 (*MYB103*, *SND1*, and *SND3*) [59]. The coding sequence of *MYB52* was PCR-amplified from wild-type cDNA (primers in Additional file 3) and cloned into pDONR221. After confirmation of sequence identity by DNA sequencing, the entry clones were subcloned into the destination vector p2GW7 to generate the effector vectors, in which a constitutive *CaMV 35S* promoter drives the expression of the secondary cell wall transcription factor genes. The normalization vector consisted of the *CaMV 35S* promoter driving the expression of the Renilla luciferase [60].

The protoplast transactivation assay was performed as previously described [61]. Briefly, a total of 2 μg of each effector, reporter, and normalization vector was transfected into tobacco BY-2 protoplasts using a Ca^2+^/PEG solution, and the cells were incubated for 24 h in the dark before lysis and measurement of luciferase activities with the Dual Luciferase Kit (Promega). Normalization was carried out by dividing the activity of the reporter firefly luciferase with the activity of the Renilla luciferase for each transfection sample. The assay was performed with eight biological replicates, each containing 100 μL of protoplast solution (500 protoplasts per μL). As a negative control, a mock vector, in which the coding sequence of the *GUS* gene was subcloned into the destination vector p2GW7, was used instead of an effector vector.

The accession numbers for the transcription factors are *MYB46*, At5g12870; *MYB83*, At3g08500; *MYB63*, At1g79180; *MYB85*, At4g22680; *MYB103*, At1g63910; *SND1*, At1g32770; *SND2*, At4g28500; *SND3*, At1g28470; *MYB52*, At1g17950; and *KNAT7*, At1g62990.

### Quantitative (q)RT-PCR analysis

Transcript levels of selected genes were determined in stems of *cse*-*2* and wild-type plants using qRT-PCR. Seeds of both genotypes were germinated in soil and grown in short-day conditions (21 °C, 9 h of light; 18 °C, 15 h of darkness) for 8 weeks, after which they were moved to long-day conditions (21 °C, 16-/8-h light/dark cycle) to allow bolting. The top 10 cm of the inflorescence stem was harvested when plants reached around 24 cm in height. Total RNA was isolated with RNeasy Plant Mini Kit (QIAgen) and treated with DNA-free (Ambion) to remove contamination with genomic DNA, and a total of 1 μg was used as template for the synthesis of cDNA using the iScript cDNA Synthesis Kit (Bio-Rad). Samples were run in triplicate on a LightCycler 480 Real-Time SYBR Green PCR System (Roche) according to the manufacturer’s instructions. Fluorescence values were exported from the lightcycler program whereupon Ct values; normalization factors and primer efficiencies were calculated according to Ramakers et al. [62] using three reference genes: Ser/Thr protein phosphatase 2A (PP2A; At1g13320), SAND family protein (At2g28390), and At2g32170 [63]. In addition, the expression of *CSE* in seedlings 12 days after germination (DAG) of *cse*-*2 proVND::CSE* lines, *cse*-*2* mutant, and the wild type was also determined, following the same protocol described above. qRT-PCR primers are listed in Additional file 3.

### Plant growth and harvest

For the vessel-specific complementation of *cse*-*2*, plants were randomized and cultivated in short-day conditions (21 °C, 9 h of light, 120 mE s^−1^ m^−2^; 18 °C, 15 h of darkness) for 8 weeks before they were moved to long-day conditions (21 °C, 16-/8-h light/dark cycle) to allow bolting. For microscopy and histochemical analysis of lignin, plants were grown until the stem reached around 24 cm in height, while for cell wall compositional analysis and saccharification, fully senesced stems were used after determination of height and weight. For such measurements, *cse*-*2proVND7::CSE#1* (*n* = 16), *cse*-*2 proVND7::CSE#2* (*n* = 21), *cse*-*2 proVND7::CSE#3* (*n* = 17), and *cse*-*2 proVND7::CSE#4* (*n* = 16) were randomly grown together with the wild type (*n* = 18) and the *cse*-*2* mutant (*n* = 17). In a similar but independent experiment, *cse*-*2 proVND6::CSE#1* (*n* = 27), *cse*-*2 proVND6::CSE#2* (*n* = 25), and *cse*-*2 proVND6::CSE#3* (*n* = 25) were randomly grown with the wild type (*n* = 18) and the *cse*-*2* mutant (*n* = 23). The senesced main stem was chopped in 2-mm pieces prior to the preparation of cell wall residue.

### Microscopy and scoring of collapsed vessels

The analysis of vasculature integrity and lignin staining with Mäule reagent was performed as described [64]. Photographs were taken using bright-field imaging with a Zeiss AxioSkop2 microscope. The presence of collapsed vessels was visually scored in a double blind experiment, in which the genotype of each cross section was omitted to the monitoring researchers. The *irx* phenotype was quantified by dividing the number of collapsed vessels by the total number of xylem vessels within a vascular bundle. To minimize the subjectivity of the analysis, only the severe phenotype (i.e., vessels with wrinkled borders or completely squeezed) was scored. A minimum of 11 vascular bundles was analyzed per genotype (average of 17 ± 5 bundles) by inspecting stem sections of three individual plants per genotype.

### Cell wall characterization and saccharification

The preparation of cell wall residue (CWR), acetyl bromide soluble lignin quantification (*n* = 7), cellulose quantification (*n* = 7), saccharification assays (*n* = 7), and thioacidolysis (WT, *n* = 7; *cse*-*2*, *n* = 7; *cse*-*2 proVND7::CSE#1*, *n* = 7; *cse*-*2 proVND7::CSE#2*, *n* = 7; *cse*-*2 proVND7::CSE#3*, *n* = 6; *cse*-*2 proVND7::CSE#4*, *n* = 4) was performed as previously described [15, 31]. Each biological replicate represents a pool of two plants. Total amount of Glc release on a plant basis was calculated with the formula: (Glc release/DW)*Average DW of the main inflorescence stems (*n* = 17–21).

### Statistical analyses

Statistical analysis of biomass parameters and biochemical data was performed with analysis-of-variance (ANOVA) with R using the lm function. The homoscedasticity was assessed with the bartlett.test function, and, if necessary (*P* < 0.01), a Box-Cox transformation was performed prior to ANOVA. However, for *cse*-*2 proVND6::CSE* data on weight, none of the Box-Cox transformations led to sufficient homoscedasticity, slightly biasing the ANOVA results. Opposite to *CSE* expression, lignin quantification, thioacidolysis, and saccharification data, there was no restriction on the number of biological replicates to include for data on height and weight. Therefore, the latter two traits were analyzed via a more powerful nested ANOVA rather than a one-way ANOVA. Upon a significant ANOVA test result (*P* < 0.05), Tukey Honestly Significant Difference (Tukey HSD) post hoc tests were performed to reveal which groups differed for the particular trait. For the nested ANOVA, three main groups (wild type, *cse*-*2* mutant and *cse*-*2 proVND::CSE* lines) were considered, whereas all individual lines (wild type, *cse*-*2* mutant and the various *cse*-*2 proVND::CSE* lines) were taken into account for one-way ANOVA. In the case of a significant nested ANOVA model, the Tukey HSD test was not performed whenever a significant nested effect (*P* < 0.01) was evident (i.e., whenever significant differences existed between the different *cse*-*2 proVND::CSE* lines). This was the case for the *cse*-*2 proVND6::CSE* data on height. Consequently, the *cse*-*2 proVND6::CSE* #3 line was subsequently removed and a new nested ANOVA model computed (Additional files 4 and 5).

## Additional files

10.1186/s13068-016-0551-9 Expression analysis of *CSE* in 12 DAG seedlings of *cse*-*2 proVND::CSE* lines, *cse*-*2* mutant, and the wild type as determined via RT-qPCR. The normalized expression of each genotype is relative to that of the wild type. Error bars indicate the standard deviation. Differences in gene expression were assessed with one-way ANOVA. Tukey HSD’s test was used as a post hoc comparison, with statistical significance considered at the 0.05 level (n = 4).
10.1186/s13068-016-0551-9 Semi-quantitative analysis of the *irx* phenotype in stem cross sections from the wild type, *cse*-*2* mutant, and the *cse*-*2 proVND::CSE* lines after Mäule staining. A) Scoring of the *irx* phenotype in the *cse*-*2 proVND7::CSE* lines; B) Scoring of the *irx* phenotype in the *cse*-*2 proVND6::CSE* lines. The values plotted on the graph are the averages of measurements made by two independent monitoring researchers in a double blind experiment. These values were calculated by dividing the total number of collapsed vessels found in each genotype by the total number of xylem vessels found in all vascular bundles scored per genotype. A minimum of 11 and an average of 17.5 vascular bundles were scored, by inspecting sections from three individual plants per genotype. Error bars indicate the standard deviation. One-way ANOVA and Duncan’s Multiple Range Test were performed to reveal significant (*P* < 0.05) differences between the various lines, which are indicated by different letters.
10.1186/s13068-016-0551-9 List of primer sequences used for cloning and quantitative RT-PCR. Both forward and reverse primer sequences are shown in the 5′ to 3′ direction. Lower case letters indicate the sites for GATEWAY™ cloning (Invitrogen), while letters in bold indicate restriction sites for classical cloning.
10.1186/s13068-016-0551-9 Statistical analysis of data on height and weight of *cse*-*2 proVND7::CSE* lines using nested ANOVA. A Bartlett’s test was first performed to test homoscedasticity; nested ANOVA was performed to test the differences between the groups, followed by Tukey HSD as post hoc test. Numbers represent the genotypes 1 = wild type, 2 = *cse*-*2*, and 3 = *cse*-*2 proVND7::CSE* line 1 to line 4, respectively.
10.1186/s13068-016-0551-9 Statistical analysis of data on height and weight of *cse*-*2 proVND6::CSE* lines using nested ANOVA. A Bartlett’s test was first performed to test homoscedasticity; nested ANOVA was performed to test the differences between the groups, followed by Tukey HSD as post hoc test. Numbers represent the genotypes 1 = wild type, 2 = *cse*-*2*, and 3 = *cse*-*2 proVND6::CSE* line 1 to line 3, respectively.

## Abbreviations

4CL: 4-coumarate:CoA ligase
AcBr: acetyl bromide
C3H: *p*-coumarate 3-hydroxylase
C4H: cinnamate 4-hydroxylase
CAD: cinnamyl alcohol dehydrogenase
CCoAOMT: caffeoyl-CoA *O*-methyltransferase
CesA: cellulose synthase
COMT: caffeic acid *O*-methyltransferase
CCR: cinnamoyl-CoA reductase
CSE: caffeoyl shikimate esterase
CWR: cell wall residue
F5H: ferulate 5-hydroxylase
G: guaiacyl
H: *p*-hydroxyphenyl
HCALDH: hydroxycinnamaldehyde dehydrogenase
HCT: *p*-hydroxycinnamoyl-CoA:shikimate/quinate *p*-hydroxycinnamoyl transferase
irx: irregular xylem
KNAT7: arabidopsis knotted1-like 7
MYB: myeloblast
NAC: NAM, ATAF1/2 and CUC2
PAL: phenylalanine ammonia-lyase
S: syringyl
SMRE: secondary wall MYB-responsive element
SNBE: secondary wall NAC binding element
VND: vascular-related NAC domain
