# Supplementary figures and images for: Improving total saccharification yield of Arabidopsis plants by vessel-specific complementation of caffeoyl shikimate esterase (cse) mutants
Source: Biotechnol Biofuels. 2016 Jul 7;9:139. doi: 10.1186/s13068-016-0551-9 (PMC4936005; doi:10.1186/s13068-016-0551-9)

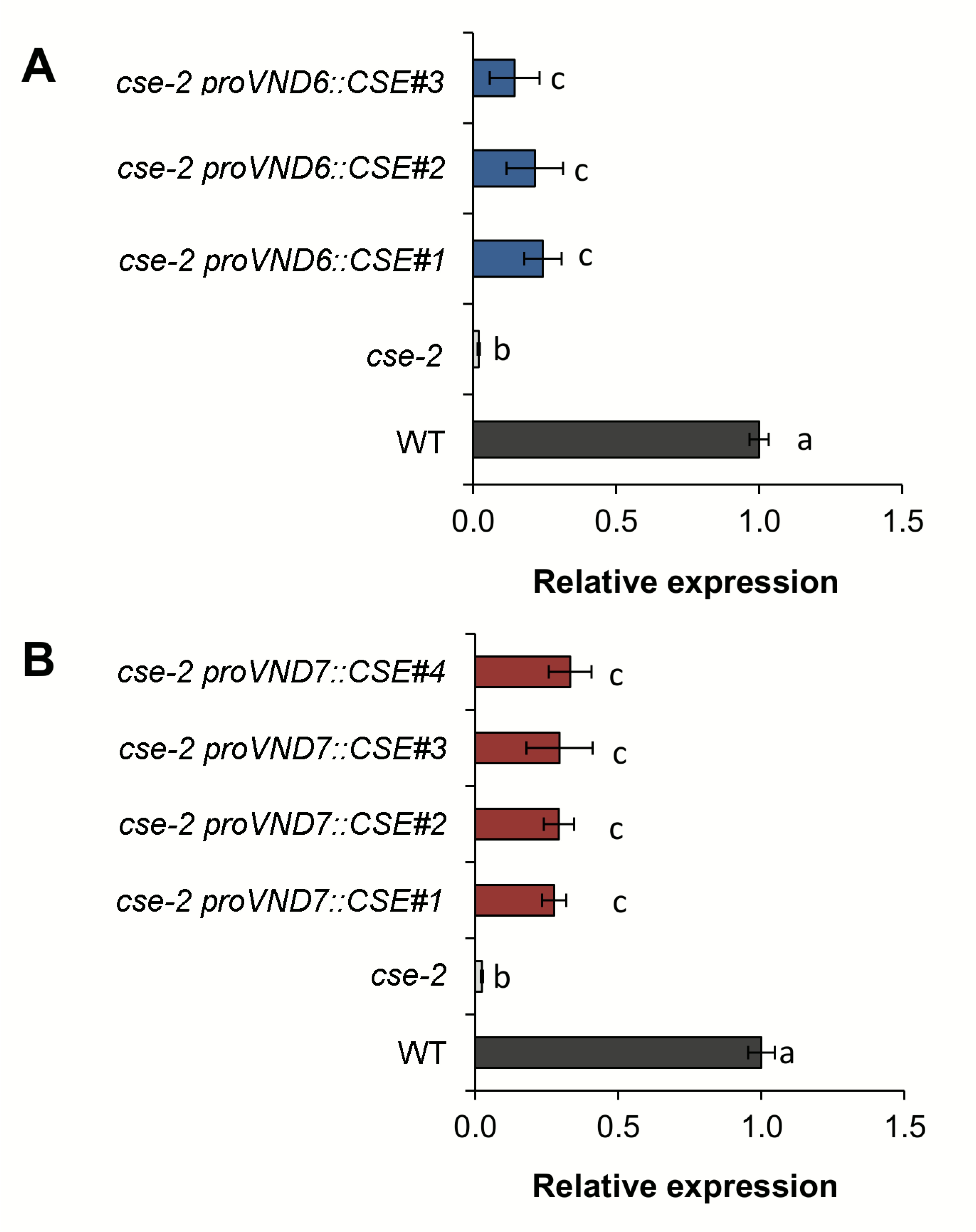

Supplement: Supplementary file 1 — 10.1186/s13068-016-0551-9 Expression analysis of CSE in 12 DAG seedlings of cse-2 proVND::CSE lines, cse-2 mutant, and the wild type as determined via RT-qPCR. The normalized expression of each genotype is relative to that of the wild type. Error bars indicate the standard deviation. Differences in gene expression were assessed with one-way ANOVA. Tukey HSD’s test was used as a post hoc comparison, with statistical significance considered at the 0.05 level (n = 4). [file 13068_2016_551_MOESM1_ESM.jpg]

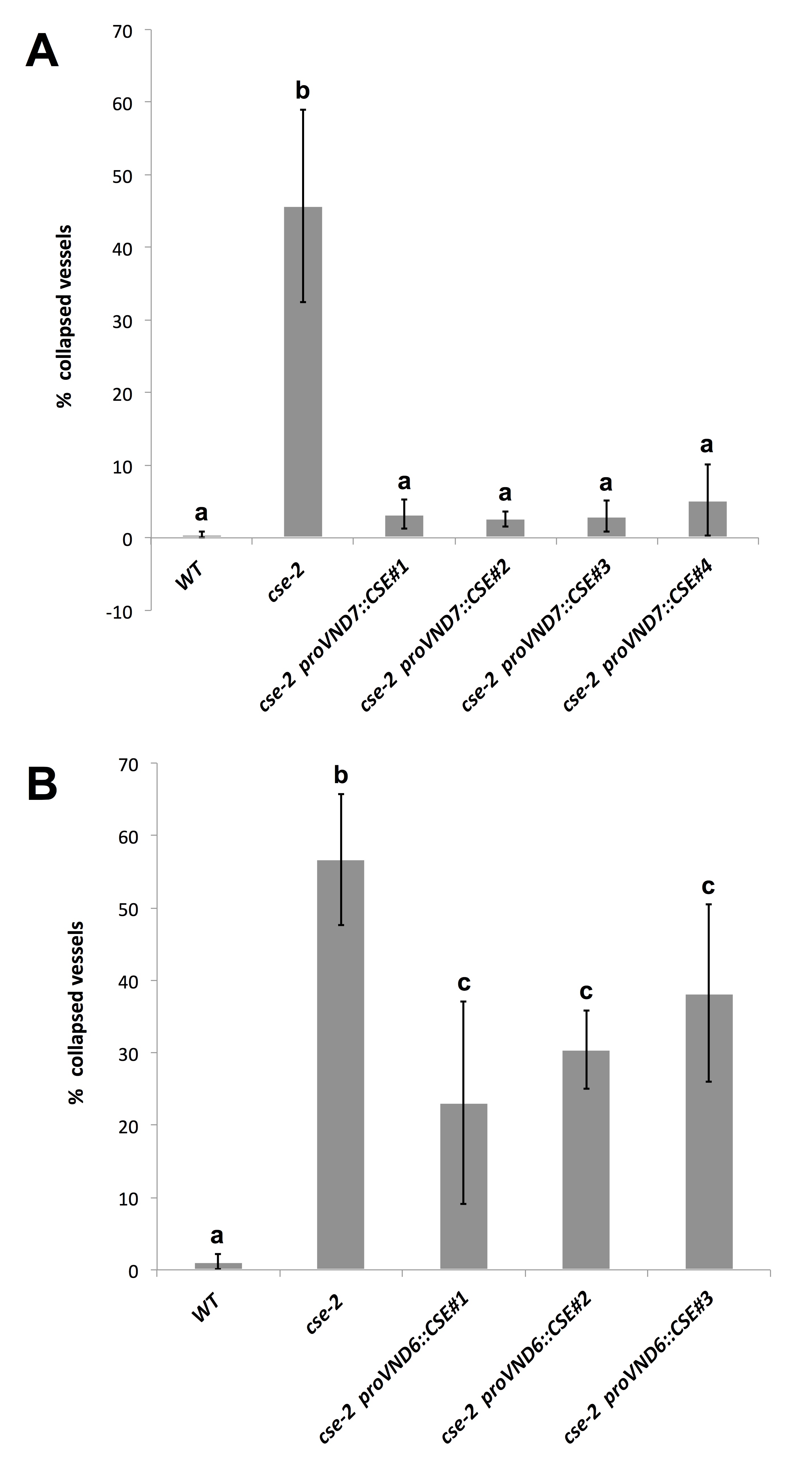

Supplement: Supplementary file 2 — 10.1186/s13068-016-0551-9 Semi-quantitative analysis of the irx phenotype in stem cross sections from the wild type, cse-2 mutant, and the cse-2 proVND::CSE lines after Mäule staining. A) Scoring of the irx phenotype in the cse-2 proVND7::CSE lines; B) Scoring of the irx phenotype in the cse-2 proVND6::CSE lines. The values plotted on the graph are the averages of measurements made by two independent monitoring researchers in a double blind experiment. These values were calculated by dividing the total number of collapsed vessels found in each genotype by the total number of xylem vessels found in all vascular bundles scored per genotype. A minimum of 11 and an average of 17.5 vascular bundles were scored, by inspecting sections from three individual plants per genotype. Error bars indicate the standard deviation. One-way ANOVA and Duncan’s Multiple Range Test were performed to reveal significant (P < 0.05) differences between the various lines, which are indicated by different letters. [file 13068_2016_551_MOESM2_ESM.jpg]
